# Optical Coherence Tomography as a Non-Invasive Tool for Plant Material Characterization in Agriculture: A Review

**DOI:** 10.3390/s24010219

**Published:** 2023-12-30

**Authors:** Sm Abu Saleah, Shinheon Kim, Jannat Amrin Luna, Ruchire Eranga Wijesinghe, Daewoon Seong, Sangyeob Han, Jeehyun Kim, Mansik Jeon

**Affiliations:** 1 ICT Convergence Research Center, Kyungpook National University, Daegu 41566, Republic of Korea; 2 Institute of Biomedical Engineering, Kyungpook National University, Daegu 41566, Republic of Korea; 3 School of Electronic and Electrical Engineering, College of IT Engineering, Kyungpook National University, 80, Daehak-ro, Buk-gu, Daegu 41566, Republic of Korea; 2021325169@knu.ac.kr (J.A.L.);; 4 Department of Electrical and Electronic Engineering, Faculty of Engineering, Sri Lanka Institute of Information Technology, Malabe 10115, Sri Lanka

**Keywords:** optical coherence tomography, agriculture, optical imaging, image processing, image analysis, disease detection

## Abstract

Characterizing plant material is crucial in terms of early disease detection, pest control, physiological assessments, and growth monitoring, which are essential parameters to increase production in agriculture and prevent unnecessary economic losses. The conventional methods employed to assess the aforementioned parameters have several limitations, such as invasive inspection, complexity, high time consumption, and costly features. In recent years, optical coherence tomography (OCT), which is an ultra-high resolution, non-invasive, and real-time unique image-based approach has been widely utilized as a significant and potential tool for assessing plant materials in numerous aspects. The obtained OCT cross-sections and volumetrics, as well as the amplitude signals of plant materials, have the capability to reveal vital information in both axial and lateral directions owing to the high resolution of the imaging system. This review discusses recent technological trends and advanced applications of OCT, which have been potentially adapted for numerous agricultural applications, such as non-invasive disease screening, optical signals-based growth speed detection, the structural analysis of plant materials, and microbiological discoveries. Therefore, this review offers a comprehensive exploration of recent advanced OCT technological approaches for agricultural applications, which provides insights into their potential to incorporate OCT technology into numerous industries.

## 1. Introduction

Plant diseases reduce production levels and cause direct and indirect major economic losses in agriculture and forestry. Pathogens and other agents, such as insects, animals, and weeds, cause direct crop losses that range from 20% to 40% of worldwide agricultural production [1,2,3,4]. In the United States, the approximate annual cost of crop damage caused by plant pathogens was about USD 33 billion [5]. Fruit diseases can also lead to huge productivity and quality losses during the harvesting and post-harvesting periods [6,7,8]. Moreover, seeds can harbor a wide range of microflora, including fungi, bacteria, nematodes, viruses, and other organisms that can cause crop diseases and cause massive crop losses [9,10]. Therefore, plant-, fruit-, and seed-borne diseases cause huge economic losses collectively, and, in most cases, the diseases can only be treated and controlled once the symptoms are detected at an advanced stage [11,12].

Several approaches for the early diagnosis of leaf diseases have been established. In the early stages of infection, visual inspection is often utilized; however, it is subjective, inefficient, time-consuming, and labor-intensive [13,14,15]. Plant diseases can also be identified using physiological, biological, serological, or molecular testing, as well as a variety of laboratory-based techniques [16,17,18,19]. Polymerase chain reactions (PCRs), enzyme-linked immunosorbent assays, and histological sectioning are some of the most common laboratory test-based, complex, time-consuming, destructive, and expensive plant disease inspection methods [20,21]. To compensate for the abovementioned limitations in plant disease diagnosis, non-invasive approaches, such as image processing [22,23,24,25], microfocus X-ray fluorescence [26], terrestrial laser scanning [15], spectroscopy [27], sonic tomography [17], GanoSken technology [28], and the electronic nose [29] have gained much popularity. However, these techniques have several limitations, including the necessity of a lengthy setup procedure, complexity, high cost, sensitivity to environmental changes, limited selectivity, and the necessity of highly sophisticated software [19,30]. X-ray [31], magnetic resonance imaging (MRI) [32], ultrasound [33], and positron emission tomography (PET) [34] imaging methods have been used to accomplish the non-invasive morphological and structural imaging of plant materials. However, these imaging techniques are limited by low image resolution and long acquisition times [35,36,37,38]. However, early disease detection in the agricultural context is still a challenge; therefore, a non-invasive optical image acquisition technique can offer a solution for early disease detection in several agricultural industries.

OCT seems to be a promising technique in plant material characterization, which is based on low-coherence interferometry. OCT is an ultra-high-resolution and purely non-invasive imaging technology that employs a non-ionizing broadband light source, which aids in the prevention of radiation-induced tissue damage [39]. The image resolution of OCT is 1–15 μm, which is 10 to 100 times better than that of ultrasound [40]; therefore, it is easy to detect a defect in the plant early by interpreting cross-sectional and three-dimensional (3D) images of soft and hard tissue, owing to this high resolution. The high-speed data acquisition capacity of OCT allows real-time imaging of in vitro, ex vivo, and in vivo samples, where structural changes can be assessed quantitatively and qualitatively. As a result of possessing the aforementioned qualities, OCT has proven to be useful in various fields of study, including dermatology [41], medical diagnosis [42], dentistry [43], tissue imaging [44], agriculture [45], entomology [46], and industrial applications [47]. Since an OCT imaging depth of 1.5 to 2 mm is sufficient for the inner-structure visualization of plant material on a micrometer scale, OCT-based agricultural studies have established a strong platform from which to confirm the applicability of OCT in plant material assessment [45,48,49,50].

In this review, six different agricultural areas are focused on, wherein OCT is employed as a powerful tool for disease detection and monitoring techniques. The contents of OCT-based inspection are divided into sections and discussed in six different chapters. In Chapter 1, the non-invasive virus screening of seed specimens through OCT imaging is reviewed. In Chapter 2, optical inspection for the detection of leaf spot diseases is reviewed. In Chapter 3, the diagnosis of physiological diseases of fruit specimens using OCT is reviewed. In Chapter 4, the optical sensing-based germination rate assessment of plant seeds is reviewed. In Chapter 5, a backpack-mounted or wearable OCT system for on-field inspection is reviewed. In Chapter 6, optical coherence imaging-based microbiological findings are reviewed. Furthermore, the future directions of OCT for agricultural applications have also been discussed in this review.

## 2. Applicational Overview of OCT in Agriculture

**Figure 1:**
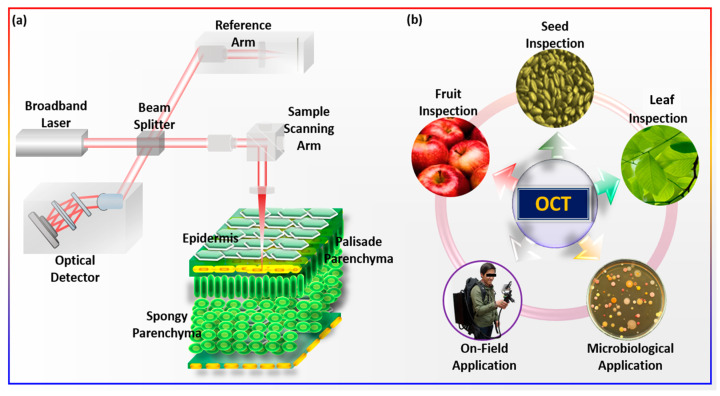
The schematic diagram represents an application overview of OCT in agriculture. (**a**) System schematic of optical coherence tomography. (**b**) OCT applications in agriculture.

OCT is a relatively new, non-invasive, non-contact, high-resolution imaging system for in vivo imaging, which is based on the Michaelson interferometer. OCT operates similarly to ultrasonic imaging, except it relies on the concept of light scattering rather than sound [51]. In OCT imaging, the variations in path length differences of the backscattered light from the different layers of the sample structure are measured to produce two-dimensional (2D) cross-sectional images of the sample structure [39]. Since light travels through air at a very high speed, it is difficult to evaluate backscattered signals directly; therefore, correlation or interferometry techniques are needed. The low-coherence interferometry method is the one that is most frequently used to determine the time delay of backscattered light. By using a known light path as a reference, the time delay or path length difference between the backscattered light path reflected from the sample and the known reference path can be calculated, which demonstrates the structural difference between the reference objects and the sample. The Michelson interferometer is the most frequently used interferometry method for OCT, whereby the light from a source is directed onto a prism or a beam splitter, or onto a fiber coupler, and is then delivered separately to the reference and sample arms. Both the light reflected from the mirror of the reference arm and the sample arm interfere at the beam splitter or fiber coupler. The final interference signal is detected by a photodetector, which creates dark and bright fringes according to the sample structures. To enable raster scanning of the sample, two galvanometric scanners are mounted on the sample arm. The lateral resolution of the OCT system depends on the used wavelength and beam optics, whereas the axial resolution depends on the coherence length and bandwidth of the light source being used. Therefore, high-resolution cross-sectional images of the sample structure can be obtained by utilizing a broad-bandwidth source with low coherence and an appropriate beam optics configuration. Figure 1a shows a schematic of the OCT system’s working principle and Figure 1b shows the application of OCT in various fields.

Fourier-domain OCT (FD-OCT) is the most common currently used technology in agriculture, which is seen in two approaches: photodetector-based swept-source OCT (SS-OCT) and spectrometer-based spectral-domain OCT (SD-OCT) [52]. SD-OCT uses a wideband laser source coupled with a spectrometer, while SS-OCT employs a frequency-sweeping laser source coupled with a photodiode to split the interference signal into single wavelengths [53]. OCT also uses the same terminology as ultrasound for single-point scans (A-Scan), linear scans (B-Scan), and area scans (C-Scan) [54]. A depth profile of the backscattering is produced along a line that is perpendicular to the object surface (A-scan) after the Fourier transform of the received signal. Then, 2D cross-sectional images are obtained via point-by-point scanning of the OCT beam passing over the material (B-scans). A set of 2D cross-sectional images is then made via line-by-line scanning of the sample, from which a 3D image stack of the sample can be created.

## 3. A-Scan Profiling for Assessing OCT Images

**Figure 2:**
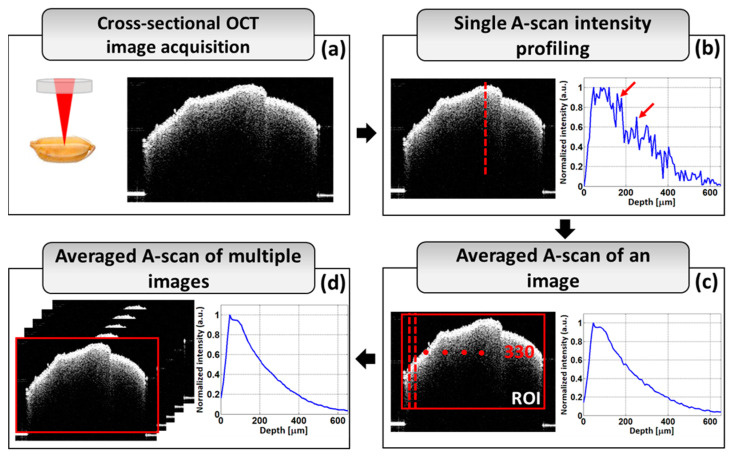
A-scan analysis for assessing OCT images. (**a**) The cross-sectional OCT image acquisition process. (**b**) Single A-scan intensity profiling. (**c**) Average A-scan of an image. (**d**) Average A-scan of multiple images.

The A-scan (amplitude scan) profiling algorithm is the most widely used technique for characterizing plant materials by assessing OCT images. Figure 2 shows how the A-scan profiling algorithm is used to obtain depth directional intensity from obtained OCT cross-sectional images. Figure 2a shows the optical imaging process for OCT imaging and a 2D cross-sectional image of the sample. The acquisition technique for a single A-line intensity profile is shown in Figure 2b; the red dotted line indicates the scanning position. Multiple irregular intensity peaks (marked by the red arrows) can be seen in a single A-line intensity profile. In Figure 2c, a total of 330 A-scan signals from the region of interest (ROI) were added together and averaged in order to eliminate the irregular intensity peaks seen in the depth intensity profile plots and to cover the whole width of the image. The averaged depth intensity profile of a single OCT image is shown in Figure 2c. In Figure 2c, the red box indicates the region of interest (ROI) of the A-scan profiling from a single OCT cross-sectional image of a sample. The number 330 indicates the number of A-scan signals that are obtained from the whole ROI of a single OCT cross-sectional image. Multiple 2D cross-sectional images of a single sample are subjected to depth scan analysis using the same technique to provide a smoother and more reliable intensity profile from an area of the sample. Figure 2d shows the averaged A-scan intensity profile for multiple OCT cross-sectional images of a sample.

## 4. Chapters

### 4.1. Chapter 1—Non-Invasive Screening for Disease in Plant Seed Specimens

**Figure 3:**
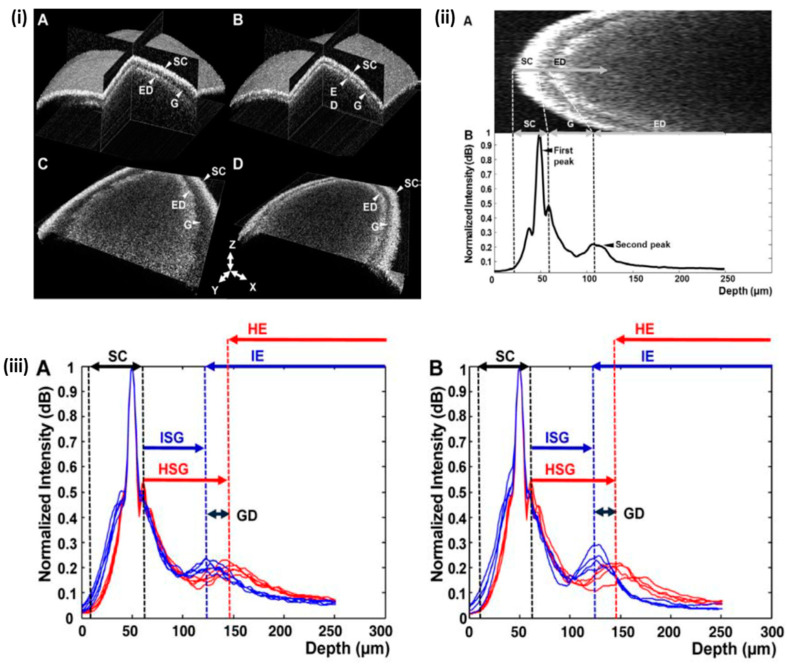
Comparison of healthy and CGMMV-infected cucumber seeds through OCT images and A-scan profiles: (**i**) 3D and en-face images (figure source [55]). (**i**(**A**,**B**)) three dimensional OCT images, and (**i**(**C**,**D**)) is the XY plane images of the healthy and infected cucumber seeds. (**ii**) OCT en-face images of cucumber seed and the corresponding A-scan profile (figure source [55]). (**iii**) The difference between healthy and CGMMV-infected seeds after heat-drying (**A**) or water immersion (**B**). SC, seed coat; ISG, infected seed gap; HSG, healthy seed gap; IE, infected seed endosperm; HE, healthy seed endosperm; GD, gap distance between healthy and CGMMV-infected seeds (figure source [55]).

The use of healthy seeds is the first and most important condition for agricultural productivity. The use of healthy seeds after separating out diseased seeds increases production and prevents huge economic losses. Optical coherence tomography can be used to detect abnormalities in the seeds and monitor morphological variations that are infected with viruses. Cucumber green mottle mosaic virus (CGMMV)-infected unhealthy cucumber seed specimens have also been distinguished from healthy seeds using OCT [55]. Seung-Yeol Lee et al. confirmed that the CGMMV-infected seeds had a narrow gap between the seed coat and endosperm compared to the healthy seed by analyzing OCT cross-sectional images and 3D volume images. Additionally, A-scan analysis was also employed to verify the result, whereby a narrow gap was found in the inner structure of the infected seeds. Figure 3i shows a 3D image of healthy and infected seeds, which have been compared from various directions, and it can be observed that the infected seeds have a narrow gap throughout the entire inner surface, unlike healthy seeds. Figure 3i(A,B) depicts a gap between the seed coat and endosperm, affecting the overall interior structure of healthy and infected seeds in the XZ and YZ planes of the 3D images. Additionally, when comparing the infected seeds in Figure 3i(D) with the healthy seeds in Figure 3i(C), a clear, narrow gap can be noticed.

As per the work of Bennett [56], the presence of the second layer in abnormal seeds can be described as one of the most distinctive interactions between plant viruses and their hosts, which is the great defense of embryos against viruses infecting the mother plant. The morphological changes in normal and infected seeds were observed using OCT images, and a distinct layer under the surface was identified by evaluating the infected seed images. To identify the anthracnose (fungus)-infected tomato seeds and evaluate its effect, Bharti et al. [57] used full-field optical coherence tomography. They measured the healthy seed coat thickness as 28.2 µm, which disappeared with infection, and the gap between the surface and endosperm was barely noticeable. The 3D-OCT (three-dimensional OCT) image revealed that the permeability of the seed coat was also affected by the infection, which plays a vital role in protecting the seed. The healthy and infected seed coat surfaces showed significant differences in the cross-sectional, en-face, and 3D images, and the two boundary layers also disappeared with infection. Yang Zhou et al. investigated mold contamination in maize kernels at an early stage using OCT [58]. They used the surface and near-surface information from the OCT images of maize kernels to analyze those changes caused by mold contamination that can be detected by feature extraction or image-processing methods. High-resolution 2D tomographic images of the microstructure of maize can be obtained via the OCT technique, and the experimental results suggest that mold-contaminated maize kernels can be identified and marked at an early stage by monitoring the near-surface layers.

### 4.2. Chapter 2—Optical Sensing-Based Germination Rate Assessment for Plant Seeds

**Figure 4:**
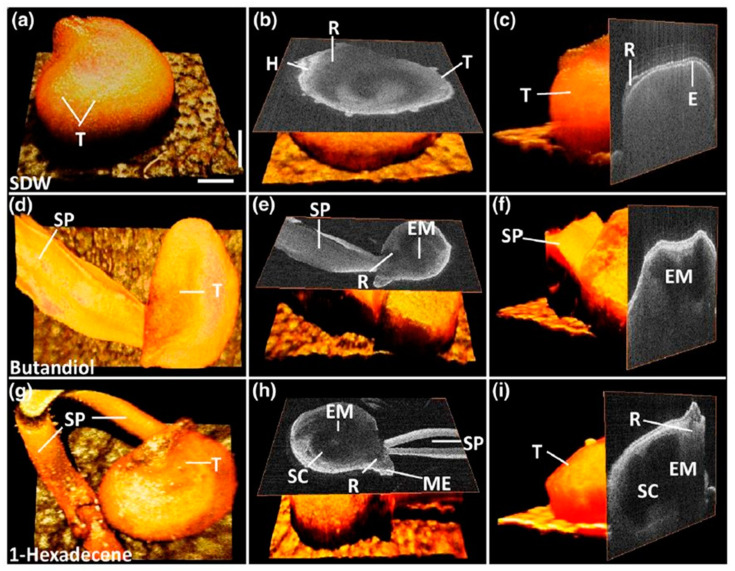
A 3D illustration of the changes in seed morphology as a result of germination: (**a**–**c**) 3D, en-face, and cross-sectional OCT images of a seed treated with SDW; (**d**–**f**) 3D, en-face, and cross-sectional OCT images of a seed treated with butanediol; (**g**–**i**) 3D, en-face, and cross-sectional OCT images of a seed treated with 1-hexadecene. C, cotyledons; E, endosperm; EM, embryo; H, hypocotyl; ME, micropylar endosperm; R, radicle; SC, storage cotyledons; SP, sprout; T, testa (seed coat). The horizontal and vertical scale bars are 700 μm and 200 μm, respectively (figure source [59]).

Seed germination rates are influenced by various chemical treatments, and OCT can be used to observe the early morphological and structural changes occurring under the seed surface. OCT was used to evaluate the germination rate acceleration of chemically primed seeds using three chemical compounds: sterile distilled water (SDW), butanediol, and 1-hexadecene [59]. In the samples treated with 1-hexadecene, morphological changes in the embryo, storage cotyledons, radicle, and micropylar endosperm, along with a long sprout, were identified within a short period in comparison with the seeds treated with other chemicals. This confirmed the suitability of 1-hexadecene as a growth-promoting chemical. OCT can visualize morphological changes in the micropylar endosperm and the development of cotyledons, embryos, and storage cotyledons, as well as help analyze germination time and measure the optimum growth-promoting chemical that will help to extend the harvesting speed. Figure 4 reveals the three-dimensional top view, the en-face view of the seed middle region, and an ortho-sliced cross-sectional view of seeds with a morphological variation that were treated with sterile distilled water (SDW) (Figure 4a–c), butanediol (Figure 4d–f), and 1-hexadecene (Figure 4g–i), respectively. The resulting en-face images demonstrate the morphological variations caused by the growth-promoting substances.

Danyang Li et al. proposed the use of biospeckle optical coherence tomography (bOCT) to investigate the response of Kaiware daikon seeds under exposure to a simulated acid mine drainage environment at different concentrations under various treatments [60]. They claimed that low pH can have a significant effect on the earlier stages of germination using bOCT. It was also found that bOCT could be used to observe the changes in seed internal activity after only 1 h when the seeds were under acid mine drainage (AMD) stress. The bOCT images clearly distinguished the changes at different concentrations of AMD treatments within a short time; the variation was found to be statistically significant and could reflect the internal activity of the seeds.

Xinhua Li et al. monitored and characterized the entire seed germination process of pea seeds to assess the morphological changes in the specimens, both externally and internally, using a web camera and OCT system, respectively [61]. A-scan analysis was applied to three different positions of the OCT cross-sectional images, which were acquired with time intervals of three hours during the germination process. In addition, seed-coat thickness was also measured from the obtained OCT cross-sectional images through A-scans.

**Figure 5:**
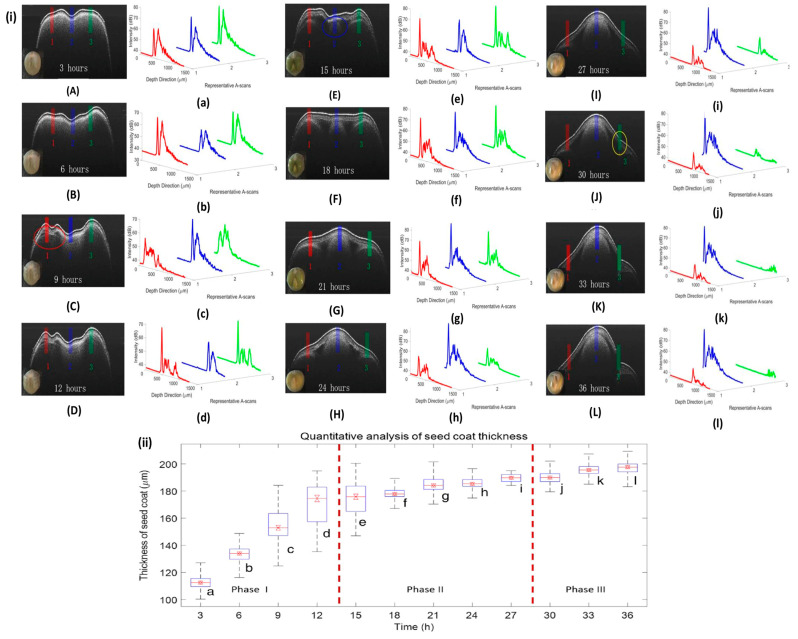
OCT and A-scan profiles for revealing the different phases of the germination at an interval of 3 h. (**i**) OCT images of the germination phases. Images (**i**(**A**–**D**)), (**i**(**E**–**I**)), and (**i**(**J**–**L**)) show phases I and III, and their corresponding A-scan profiles (**i**(**a**–**d**)), (**i**(**e**–**i**)), and (**i**(**j**–**l**)), obtained from positions 1, 2, and 3, are marked by the red, blue, and green colors, respectively (figure source [61]). The cotyledon layer becomes visible in (**i**(**C**)) marked by red circle; the radicle first observed in (**i**(**E**)) marked by blue circle; the seed coat cracked in (**i**(**J**)) marked by yellow circle. (**ii**) Boxplot for the thickness of the seed coat at different germination phases, where (**ii**(**a**–**l**)) represent the corresponding data for the A-scan shown in (**i**(**a**–**l**)) (figure source [61]).

The seed coat absorbs water from the beginning of the imbibition phase (phase I) until it reaches the lag phase, and Figure 5i(A–D) depicts this process. A large proportion of air pores exists in the early stage of phase I in the seed coats and cotyledons, resulting in a strong scattering OCT signal. Figure 5i(a–d) shows the A-scan profile of the obtained OCT images, where the ROI is marked by 1, 2, and 3 of the red, green, and blue lines. The water intake modifies the internal inhomogeneity from the subsurface context, allowing light to pass through to the deeper tissues. These changes are revealed by the reduced signal intensity of the endocarp layer and in the appearance. During phase II, the water level of the seeds remains relatively constant, while metabolic activities rise, as shown in Figure 5i(E–I); the corresponding A-scan is shown in Figure 5i(e–i). As the young seedling establishes itself, in phase III (final phase) an increase in water intake can be detected, as shown in Figure 5i(J–L), and the corresponding A-scan is shown in Figure 5i(j–l). Figure 5ii illustrates the boxplot of the maximum whisker length, which is specified as being two times the interquartile difference. The boxplot of the maximum whisker length, which is defined as two times the interquartile difference, is shown in Figure 5ii. A horizontal line within each box denotes the median of the seed coat, while the box’s bottom and top margins denote the first and third quartiles, respectively. As heavy metals considerably affect seed germination and plant growth, bOCT was used to examine the effects of increasing Zn concentrations on lentil seed germination and seedling growth by Y. Sanath K. De Silva et al. [62]. After exposing each sample to different Zn concentrations, the bOCT intensity was recorded at 0, 6, 12, and 24 h to observe the effect of different Zn concentrations on the internal activity of seed specimens. Due to the micronutrient impact of Zn at low concentrations, the samples treated with 5 mg/L and 10 mg/L Zn had greater internal activity compared to the control, whereas the internal activity of the sample treated with 100 mg/L was found to be considerably lower compared to other samples, due to the toxic effect of Zn at high concentrations.

### 4.3. Chapter 3—Optical Inspection for the Detection of Leaf Spot Diseases

**Figure 6:**
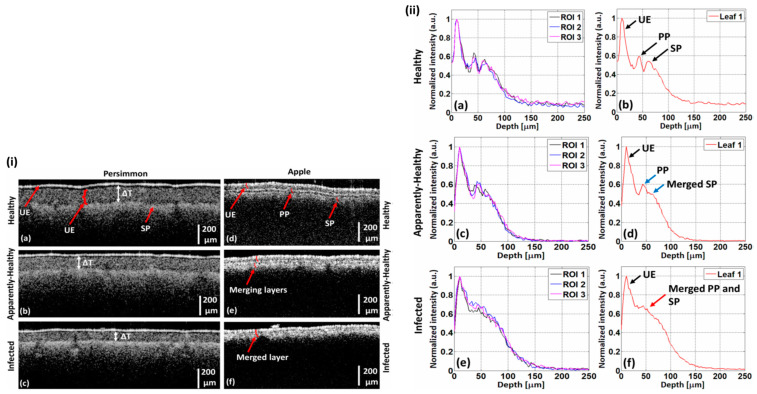
Comparison of healthy, apparently healthy, and infected leaves examined using OCT cross-sectional images and A-scan profiles. (**i**) The 2D cross-sectional images of healthy, apparently healthy, and infected persimmon and apple leaves. (**i**(**a**–**c**)) OCT cross-sectional images of persimmon leaves. (**i**(**d**–**f**)) OCT cross-sectional images of apple leaves (figure source [64]). (**ii**) Depth profiles of healthy, apparently healthy, and infected apple leaves; (**ii**(**a**,**b**)), (**ii**(**c**,**d**)), and (**ii**(**e**,**f**)) show the depth profiles of healthy, apparently healthy, and infected apple leaves, respectively. (**ii**(**a**,**c**,**e**)) Depth intensity profiles of three regions of interest (ROIs) from a single leaf. (**ii**(**b**,**d**,**f**)) The averaged depth intensity profiles of three ROIs from a single leaf (figure source [64]).

OCT imaging reveals the specific infected area of an abnormal leaf, as well as the strength and direction of fungus activity, which can be useful in preventing fungal disease in apple trees. Tzu H. Chow et al. diagnosed virus infection in orchid plants using high-resolution OCT [63]. In their study, OCT was used to identify highly scattering top and lower epidermal layers in the leaves of virus-infected plants that are invisible under histological examination. Also, despite having similar visual symptoms to those seen in virus-infected plants, the high level of scattering characteristic of the epidermal layers of virus-infected leaf samples was not seen in the leaves of stressed plants. The high level of scattering in the epidermal layers suggested that the leaves were infected with Cymbidium Mosaic Virus (CymMV), as confirmed by the enzyme-linked immunosorbent assay (ELISA) test, which is the current gold standard test for detecting virus infection in orchid plants. In our previously published article, a threshold for the pre-identification of palisade parenchyma (PP) and spongy parenchyma (SP) layer anomalies in persimmon and apple-leaf specimens was defined using the depth profile approach, based on OCT cross-sections [64]. A set of OCT cross-sectional images of apple and persimmon leaves were employed to quantitatively evaluate the inner structure of the leaf specimens, where it was observed that the thickness between the PP and SP layers gradually decreased in apparently healthy leaves and merged in infected apple leaves. In the persimmon leaves, PP and SP layers were gradually decreased in both apparently healthy and infected specimens. Figure 6 illustrates the OCT cross-sectional images of healthy, seemingly healthy, and diseased persimmon and apple leaves. The UE, PP, and SP layers in the persimmon leaves are clearly visible in Figure 6i(a–c), and the thickness difference between the layers is indicated by white arrows. Similar cross-sectional images of healthy, apparently healthy, and infected apple leaves are shown in Figure 6i(d–f).

**Figure 7:**
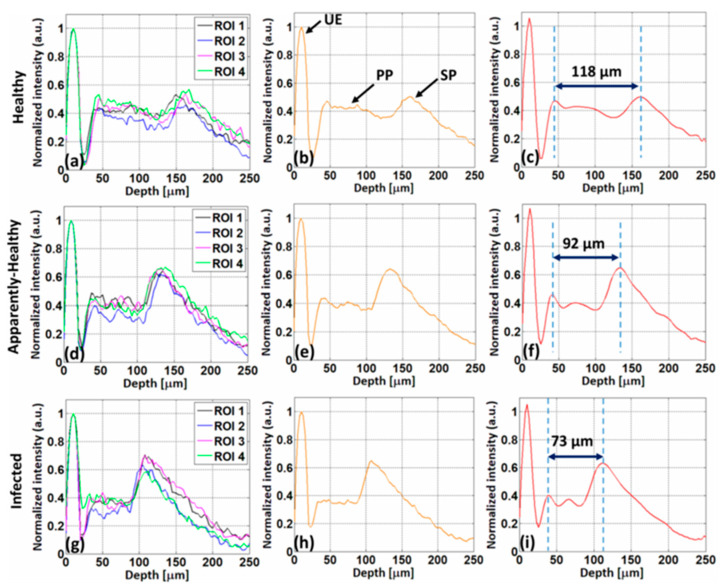
Comparison of healthy, apparently healthy, and infected persimmon leaves using A-scan profiles. (**a**–**i**) A-scan profiles of healthy, apparently healthy, and infected leaves, respectively. (**a**,**d**,**g**), (**b**,**e**,**h**), and (**c**,**f**,**i**) A-scan profiles of four ROIs, average depth profiles of four ROIs, and curve-fitted depth profiles of four ROIs of a single persimmon leaf, respectively. UE: upper epidermis, PP: palisade parenchyma, SP: spongy parenchyma (figure source [64]).

The layers become merged in the infected leaves rather than the healthy and apparently healthy leaves, which is shown in Figure 6i(e,f), respectively. Figure 6ii(a–f) displays the depth intensity profiles of healthy, apparently healthy, and infected apple leaves, respectively. Figure 6ii(a,c,e) shows the depth intensity profiles of three ROIs from a single apple leaf as black, blue, and magenta, respectively. The average depth intensity profiles of three ROIs from single healthy, apparently healthy, and infected apple leaves are shown in Figure 6ii(b,d,f), respectively. The peaks are apparent in Figure 6ii(b) and, gradually, the intensity decreases in the infected leaves, which is shown in Figure 6ii(d,f). In Figure 7 the depth intensity profiles of healthy, apparently healthy, and infected persimmon leaves are presented, respectively. The four ROIs of a single persimmon leaf’s depth intensity profiles are represented by the colors black, blue, magenta, and green. Figure 7a,d,g shows the depth intensity profiles, Figure 7b,e,h shows the average depth intensity profiles, and Figure 7c,f,i shows the curved fitted thickness intensity profiles of four ROIs from a single healthy, apparently healthy, and infected persimmon leaf, respectively. Rapid plant disease diagnosis using an image processing-based artificial intelligence system was demonstrated recently by Sanjaya Shankar Tripathy et al. [65], wherein image acquisition, pre-processing, segmentation, feature extraction, statistical analysis, classification, and disease diagnosis processes were employed.

The growth and spread of the leaf rust disease caused by *Puccinia triticina* in wheat leaves were assessed in a study by Adya Rateria et al., where the OCT images of epidermal and parenchyma cell layers were correlated with the histological images to show the distinctive leaf morphological boundaries [48]. A-scan analysis was used to monitor and compare the morphological changes in the infected leaf with a healthy leaf.

However, besides assessing leaf disease through OCT inspection, several OCT applications regarding plant leaves were also demonstrated in various previously published studies for different purposes. In one, a bOCT has been applied to monitor short-term activity changes during the foliar application of phytohormones to a plant. Different concentrations of the plant growth hormone gibberellic acid (GA_3_) were applied to the leaves of Chinese chives and their effects were monitored through bOCT contrast images, where OCT structural images had failed to show any differences [66]. Biospeckle OCT imaging was also applied to the leaves of Chinese chives to assess the response of the subjected leaves to ozone (O_3_) stress at different concentrations [67]. The standard deviation of the biospeckle signal from the back and front surfaces of the leaves was calculated, where both surfaces were found to show an increased fluctuation in the biospeckle signal under O3 stress. Furthermore, the internal cell structure of the leaves could be distinguished in the OCT biospeckle images, while it was not clearly visible in traditional OCT cross-sectional images.

### 4.4. Chapter 4—Diagnosis of Physiological Diseases of Fruit Specimens

**Figure 8:**
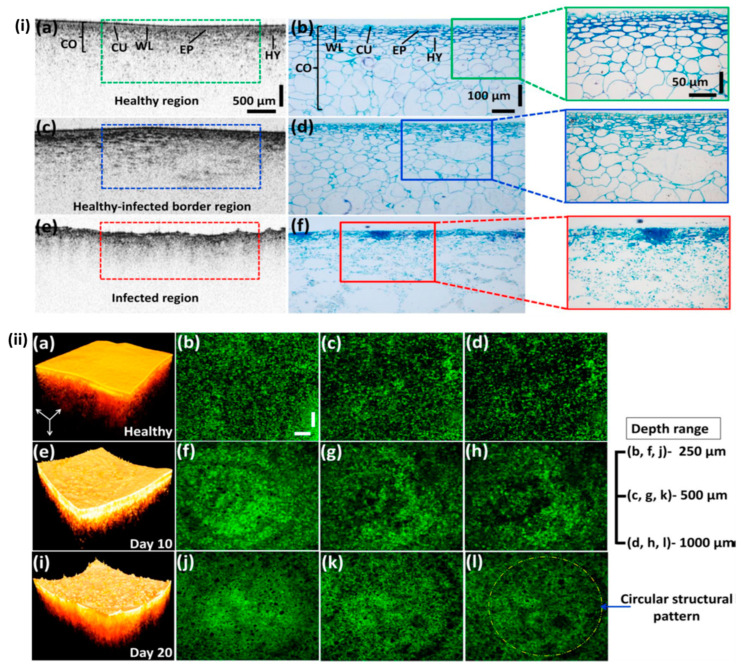
Illustration of the structural differences in fruit specimens through histology, OCT cross-section, and en-face images with the progression of the disease. (**i**) Histological validation of the 2D OCT images obtained from healthy and naturally infected fruit specimens. (**i**(**a**,**b**)) Morphology of healthy fruit. (**i**(**c**,**d**)) Morphology of apparently healthy fruit. (**i**(**e**,**f**)) Morphology of entirely infected fruit (figure source [49]). (**ii**) Illustration of the inner structure of healthy fruit and the changes in its structure at the depth direction, using en-face images. (**ii**(**a**–**d**)) The 3D and en-face images of healthy specimens at depth direction. Images (**ii**(**e**–**l**)) illustrate the morphological changes in depth direction with disease progression (figure source [49]).

One of the most damaging diseases in apple production is bitter rot, which is caused by *Colletotrichum* spp. [68]. OCT was employed to characterize bitter-rot progression on apple specimens [49]. The goal of the study was to determine the initial rotting status in apple specimens in the early stages by using 2D-OCT images, intensity profiles, and circular tissue pattern formations in both en-face OCT and boundary detection analyses. The visually non-identifiable circular tissue patterns, created under the epidermal cell layer owing to early rotting, were well represented by the obtained en-face images. Figure 8i displays the histological confirmation of OCT images that were obtained from approximately the same scan levels. Figure 8i(a,b) displays the cross-sectional images, as well as the enlarged histological view of internal morphological structures that are clearly visible in healthy specimens, such as wax layers, cuticles, epidermis layers, and hypodermis layers. Figure 8i(c,d) and i(e,f) represent the cross-sections of a partially infected and entirely infected specimen, respectively. Both the histological and OCT analysis of partially infected and fully infected specimens revealed the absence of the mentioned layers and morphological boundaries, as seen in Figure 8i(c,d) and Figure 8i(e,f), respectively. Figure 8ii shows the three-dimensional analyses of the depth direction in en-face OCT images acquired at depths of 250 μm, 500 μm, and 1000 μm, respectively. In Figure 8ii(b–d), the tissue distribution is clearly visible in all depth ranges. Circular regions below the epidermis layer started to emerge as a result of the bitter-rot disease symptoms, as seen in Figure 8ii(f–h). The boundary continued to develop as a result of the expanding spots growing in the center of the infected area, as seen in Figure 8ii(j–i).

The bruise on a pear fruit was also visualized through OCT imaging and a quantitative analysis model was proposed to classify unbruised and bruised tissue automatically [69]. Following boundary detection in the OCT images, A-scan analysis was applied to determine four possible indicators for bruises in pear fruit, including the OCT signal slope, light penetration depth, shaping, and scaling, which were taken into consideration for quantitative analysis. It was demonstrated that when the pear fruit was mechanically damaged, the slope, shape, and scaling of the OCT signal decreased while the depth of light penetration increased. A fruit bruise detection technique based on inner microstructural parameters using OCT was demonstrated by Yang Zhou et al. [70,71]. In this study, total cell surface area, average cell surface area, average cell Feret diameter, equivalent diameter, and the amount of parenchyma cells from bruised and non-bruised loquat tissue were measured. The total cell surface area and the cell number in bruised and non-bruised loquats differed significantly, suggesting that these two parameters might be utilized as indicators for bruise detection.

**Figure 9:**
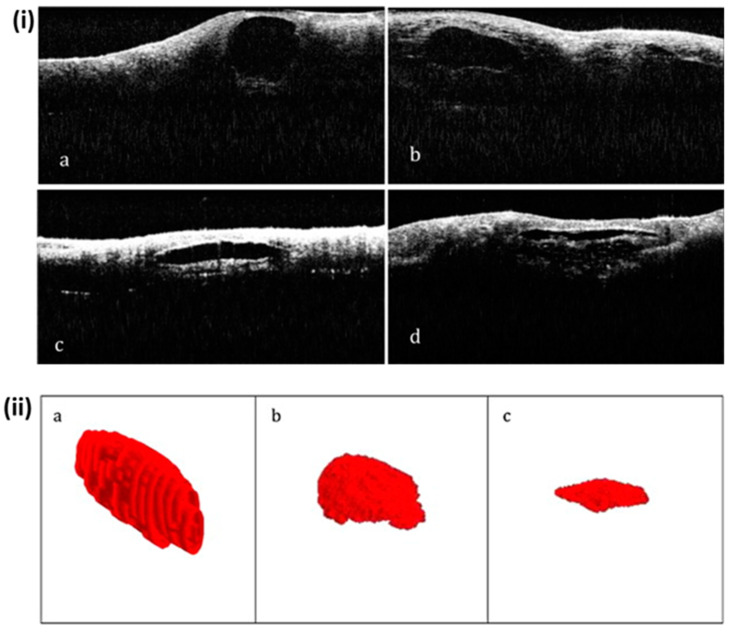
Illustration of the development of progressive rind breakdown (RBD) in mandarin fruit: (**i**(**a**)) unaffected fruit, (**i**(**b**)) mildly affected, (**i**(**c**)) moderately affected, and (**i**(**d**)) severely affected (figure source [72]). (**ii**) Oil glands in 3D representation with (**a**) no RBD, (**b**) moderate RBD, and (**c**) severe RBD (figure source [72]).

OCT was also employed to optically screen *venturia nashicola* caused pear scab disease using pear leaves and fruits, where the morphological changes of pear scab-infected Asian pear (*Pyrus pyrifolia*) leaves and fruits were assessed [72]. Here, the signal intensity difference between healthy and infected samples, owing to cross-sectional layer reduction in the infected sample, was confirmed using depth profile analysis. The histological changes associated with the progression of rind breakdown (RBD) disorder in ‘Nules Clementine’ mandarins were investigated by Magwaza et al. using OCT imaging [72]. The immediate and non-destructive acquisition of healthy and RBD-affected intact mandarin fruit images was conducted using the Thorlabs OCT system to visualize histological and microstructural features in intact rind tissue, where it was observed that the oil glands stayed intact in unaffected fruit and gradually collapsed in RBD-affected fruit, and the collapsed oil glands became increasingly deformed and flattened at an advanced stage of the disorder. Figure 9i presents illustrations of the oil glands at various phases of RBD. The unaffected fruit in Figure 9i(a) had intact oil glands with an almost round shape, whereas those in the afflicted fruit in Figure 9i(b–d) eventually collapsed. Figure 9ii(a–c) shows the 3D representations of oil glands with no RBD, moderate RBD, and severe RBD, respectively. The glands of healthy mandarins generally have an ellipsoidal form, but as the RBD progressed, they severely flattened, became irregular, and decreased in size.

### 4.5. Chapter 5—Wearable OCT for On-Field Inspection

**Figure 10:**
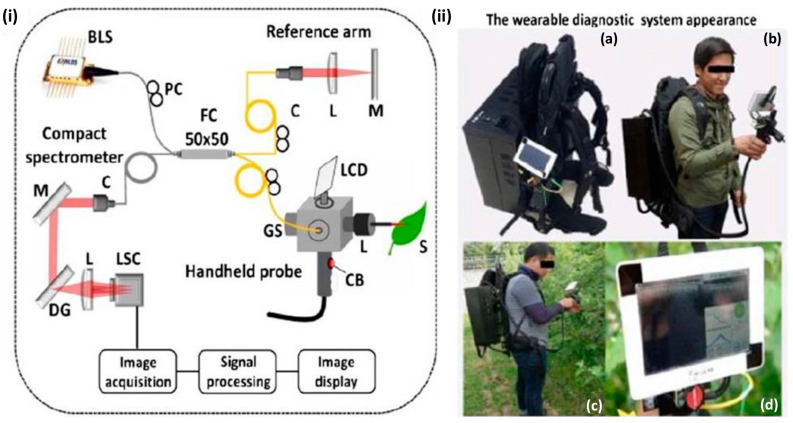
Compact, portable, and wearable OCT imaging modality configuration. (**i**) Schematic diagram of the OCT system. BLS, broadband laser source; C, collimator; CB, capture button; DG, diffraction grating; FC, fiber coupler; GS, Galvano scanner; L, lens; LCD, liquid crystal display; LSC, line scanning camera; M, mirror; PC, polarization controller; S, sample (figure source [50]). (**ii**) Wearable imaging modality appearance. (**ii**(**a**)) complete system, (**ii**(**b**)) wearable OCT with its operator, (**ii**(**c**)) wearable OCT in action, and (**ii**(**d**)) display of in vivo and real-time imaging (figure source [50]).

Our research group has developed a wearable (backpack-type) diagnostic imaging modality, employing OCT to meet the demand for non-contact inspection equipment in both indoor and outdoor experimental environments [50]. This compact, versatile backpack-type imaging system consists of a compact spectrometer, a miniature computer, a rechargeable power source, and a handheld inspection probe. To preview the 2D-OCT image, a user-friendly user interface is displayed on the miniature built-in LCD screen, and a built-in button on the handheld device is attached to save the obtained images. Therefore, the operator only has to perform three simple steps to begin the inspection while wearing the system: turning it on, previewing the acquired images, and saving the results. Through the direct use of the imaging modality in agricultural fields, this technology improves real-time in situ specimen inspection and minimizes the limitations of complex tabletop inspection modalities in the laboratory. Figure 10i shows the schematic of a conventional spectrometer-based, compact, customized, SD-OCT system for a backpack-type imaging facility, comprising a light source, reference arm, LCD monitor-oriented handheld-probe-based sample arm, spectrometer, and image processing unit. The developed wearable (backpack-type) diagnostic modality with a diagnostic procedure system is depicted in Figure 10ii, wherein a user can easily move the whole OCT system around on his back during the real-time field inspection. The full system is shown in Figure 10ii(a), the system and its operator are shown in Figure 10ii(b), the system is shown in activity in Figure 10ii(c), and the in vivo and real-time imaging system is shown in Figure 10ii(d). Field experiments were conducted in apple, persimmon, and pear plantations to evaluate the robustness, feasibility, and precise imaging capability of the developed system. On-field inspection results of the apple, persimmon, and pear plantations that were obtained using the wearable (backpack-type) diagnostic imaging modality are listed in Table 1. In the system program interface, an automated software-based OCT signal intensity detection mechanism was implemented to achieve fully automated confirmation of the healthy and infected states of leaf specimens during the real-time inspection process.

### 4.6. Chapter 6—Optical Coherence Imaging-Based Microbiological Findings

Microorganisms play a significant role in agriculture in several ways. Nitrates and other nutrients are added to the soil by microorganisms, which also maintain the soil fertility and enhance its quality [73]. Microorganisms contribute to the compositing process that results in manure [74]. Additionally, the process gives the plants specific antibiotics, nutrient content, and growth-promoting chemicals to aid in plant growth [75]. However, OCT has been applied to microorganism assessment in only a few studies.

**Figure 11:**
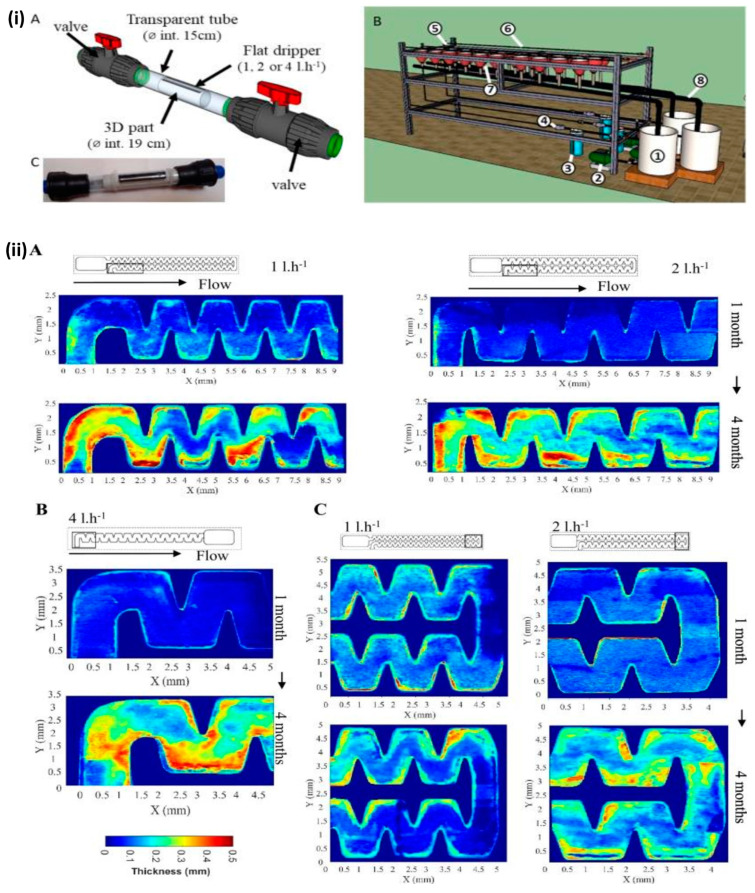
Assessment of biofilm thickness using OCT imaging: (**i**(**A**,**C**)) dripper system, (**i**(**B**)) test bench (figure source [78]) The test bench was composed of 1. a tank (60 l); 2. a water pump; 3. a 0.13 mm mesh screen filter; 4. a pressure reducer; 5. a pressure gauge; 6. the drip line with an emitter system located at 10-cm intervals; 7. a collector; 8. a gutter. (**ii**(**A**)) Measurement of biofilm thickness at the inlet of drippers. (**ii**(**B**)) Measurement of biofilm thickness in the return of drippers. (**ii**(**C**)) The return areas of drippers were measured after 1 and 4 months (figure source [78]).

Mohan et al. employed SS-OCT imaging to monitor the growth of bacteria and the continuous formation of biofilm in real time [76]. A newly developed mathematical model was used for the characterization of different bacterial colonies and biofilms by calculating backscattering optical properties, which were then verified with the conventional ‘Gram staining’ method. Obtained results revealed that Gram-positive (Bacillus lichaeniformis and Bacillus subtilis) bacteria scatter light more than Gram-negative Acrylic (BITNR004) and S3b (BITGN002) bacteria because their cell walls contain a thick coating of peptidoglycan. Real-time demonstrations of the developing links between two colonies and the migration of bacteria to establish new colonies inside the medium were also demonstrated. Moreover, the biofilm formation in drip irrigation systems was demonstrated non-invasively using OCT [77,78]. In the drip irrigation system, the zigzag structured labyrinth allowed water from the intake to pass into the basin compartment, and the transparent coverslip of the labyrinth allowed for non-invasive OCT imaging. The thickness of the biofilm was measured using OCT imaging to evaluate its formation, and it was found that the biological fouling increased with time. The dripper system is shown in Figure 11i(A,C) and it consists of a solid tube with an internal diameter of 19 mm that presses the dripper against the transparent tube’s walls and has an outlet hole punched above the outlet basin. Each of the three lines is linked to a different tank and a pump, as illustrated in Figure 11i(B), and nine drippers are connected according to the variety of drippers, using polyethylene tubing. The biofilm thickness assessed using the OCT technique at the dripper flow rates is shown in Figure 11ii. Figure 11 ii(A) illustrates the progression of biofouling thickness at the inlet of 1, 2 l·h^−1^ drippers, and Figure 11ii(B) illustrates the return dripper areas of 4 l·h^−1^ drippers after one and four months, respectively. Initially, biological fouling at the intake region was concentrated in the first baffle area and at its corners, and it subsequently extended to the succeeding corner baffles. After four months, the biofilm thickness was higher in the baffle corners for all the drippers. In both types of drippers, the large bend caused the biofilm thickness in the return area to expand the most markedly (Figure 11ii(C). The OCT imaging application in distinct agricultural fields are summarized in Table 2.

## 5. Conclusions and Future Directions

This review highlights the recently advanced OCT technological approach as a potential tool for disease detection and physiological assessment in six distinct agricultural fields. Following non-invasive OCT imaging, 2D cross-sectional, en-face, and 3D volumetric images were used to detect physiological changes in plant materials through different image analysis techniques. OCT imaging was used non-invasively to monitor germination rates and seed diseases, as well as to identify leaf diseases, assess physiological disorders in fruit specimens, and conduct on-field inspections and microbiological discoveries. The A-scan analysis applied to OCT cross-sectional images can reveal vital information about early disease diagnosis and physiological changes in plant materials. However, the traditional techniques used to evaluate the above-listed measurements have several drawbacks, including invasive inspection, complexity, extended processing times, and expensive features. OCT technology has been widely utilized to evaluate plant materials in several ways, due to its non-invasive, high-resolution, and real-time imaging capabilities. The following list outlines some further potential uses for OCT imaging in the future.

In vivo plant material inspections (on-field inspections) can be carried out in real time using the handheld probe included in the developed wearable OCT system. In future advancement of the developed wearable back-pack OCT system, the motion artifacts during the image acquisition, weight of the system, short operational time due to short battery life, high power consumption, waterproof coating of the system, and the short length of the handheld probe should be taken into account.

Many OCT systems stitch together small-area image data, using a mosaic approach to produce large-area images. However, the applicability of the mosaic approach to stitching small-area images together to make a large-area image is limited by the inconsistency of results caused by sample stage controlling inaccuracy, sample stage vibration, and additional post-processing time, where multiple images need to be captured for a single image. Therefore, a large-area-scanning OCT system might be a useful device to monitor the whole leaf area during inspection.

Consumer acceptance is hampered by postharvest softening, which is related to moisture loss and the loss of relative humidity during storage. By assessing mechanical stress, OCT can be used to evaluate crop quality in the postharvest storage period. It can also be used to observe cellular changes, which may help when producing large volumes of crops to export to distant markets and help to reduce economic loss.

OCT can be used in the context of plant-beneficial microbes (PBMs), which are growth-promoting rhizo-microorganisms that directly establish a relationship with plants. PBMs lead to the enhancement of plant growth, increase nutrient uptake, restore soil fertility, and improve plant resilience to abiotic and biotic stresses. OCT can be actively applied to assess the activity of PBMs in improving crop productivity and reducing the use of agrochemicals.

The non-destructive measurement of fluidic flow and flow velocity has already been achieved using OCT [79,80]. OCT has been widely used for the detection of blood flow and flow velocity; therefore, improvements in this field can be useful to the research of fluid dynamics in related plants. Future research may explore the possibility of studying the flow dynamics associated with plants, using highly sensitive Doppler-OCT and a double-correlation OCT-directed blood vessel spatial distribution detection approach.

Artificial intelligence (AI)-based microbiological identification and classification applications using OCT imaging can improve microbiological assessment capability in the agricultural field. However, high-quality and diverse training data are necessary for accurate AI models, which might be challenging. OCT imaging-based bacterial colony assessment using AI has not been descriptively reported to date.

## Figures and Tables

**Table 1:** Average, standard deviation, minimum, and maximum thicknesses for apple, pear, and persimmon leaves on experimental days 1, 15, and 30 (table source [50]).

Leaf Type, Experimental Day	Avg. (μm)	STD (μm)	Min. (μm)	Max. (μm)
Apple, day 1	49.42	1.03	46.42	53.25
Apple, day 15	78.62	1.72	75.96	81.86
Apple, day 30	113.05	3.21	109.72	116.85
Pear, day 1	41.31	1.12	37.98	44.88
Pear, day 15	62.74	2.24	59.08	67.65
Pear, day 30	99.39	3.12	97.06	102.15
Persimmon, day 1	228.92	8.52	215.22	243.35
Persimmon, day 15	163.69	1.93	160.36	167.47
Persimmon, day 30	120.82	1.73	118.16	124.66

**Table 2:** OCT imaging application in distinct agricultural fields.

Applications	Sample Type	Cause of Plant Material Changes	OCT-Type	Center Wavelength of the Light Source (nm)	References
Screening of disease in plant seed	Cucumber seed	CGMMV ^d^	TD-OCT	1310	[55,57,58]
	Tomato seed	Anthracnose (fungus) ^d^	FF-OCT	650	
	Maize kernels	Mold infection ^d^	SD-OCT	840	
Seed germination rate assessment	*Capsicum annum* seed	Growth-promoting chemical ^i^	SS-OCT	1310	[59,60,61,62]
	*Raphanus sativus* L. seed	Acid mine drainage ^i^	bOCT	836.1	
	Pea seed	N/A	SD-OCT	840	
	Lentil seed	Zn concentration ^i^	bOCT	836.1	
Leaf disease and morphological assessment	Wheat leaf	Fungal infection ^d^	SS-OCT	1060	[48,63,64,65,66,67]
	Orchid leaf	Virus infection ^d^	FD-OCT	820	
	Persimmon and apple	Circular leaf spot ^d^, apple blotch ^d^	SD-OCT	850	
	Wheat leaf	N/A	SS-OCT	1060	
	Chinese chive leaf	Plant growth hormone ^i^	bOCT	836.1	
	Chinese chive leaf	Exposure to ozone ^i^	SD-OCT	836.1	
Assessment of physiological disease of fruit	Apple fruit	Bitter-rot ^d^	SS-OCT	1310	[49,69,70,71,72]
	Pear fruit	Bruising	SD-OCT	1300	
	Loquat fruit	Bruising	SD-OCT	1300	
	Loquat fruit	Bruising	SD-OCT	1300	
	Mandarin fruit	Rind breakdown disorder ^d^	SD-OCT	930	
Wearable OCT for on-field inspection	Apple leaf	MarssoninaCoronaria ^d^	SD-OCT	850	[50]
OCT-based microbiological findings	Bacterial colonies and biofilms	N/A	SS-OCT	1064	[76,77,78]
	Biofilm in drip irrigation devices	N/A	SD-OCT	930	
	Milli-labyrinth channel and bacterial communities	N/A	SD-OCT	930	

^d^ Disease. ^i^ Influencer.

## Data Availability

No new data were created or analyzed in this study. Data sharing is not applicable to this article.

## Abbreviations

2D	two-dimensional
3D	three-dimensional
AMD	acid mine drainage
bOCT	biospeckle optical coherence tomography
CGMMV	cucumber green mottle mosaic virus
CymMV	Cymbidium mosaic virus
ELISA	enzyme-linked immunosorbent assay
FD-OCT	Fourier-domain OCT
GA_3_	gibberellic acid
LCD	liquid crystal display
MRI	magnetic resonance imaging
OCT	optical coherence tomography
O_3_	ozone
PCR	polymerase chain reaction
PET	positron emission tomography
PP	palisade parenchyma
ROI	region of interest
RBD	rind breakdown
SS-OCT	swept-source OCT
SD-OCT	spectral-domain OCT
SDW	sterile distilled water
SP	spongy parenchyma
TD-OCT	time-domain OCT
UE	upper epidermis
